# The Effects of *Panax ginseng* and *Panax quinquefolius* on Thermoregulation in Animal Models

**DOI:** 10.1155/2015/748041

**Published:** 2015-02-01

**Authors:** Bin Na Hong, Moon Ho Do, You Ri Her, Yeong Ro Lee, Tong Ho Kang

**Affiliations:** ^1^Department of Audiology, Nambu University, Gwangju 506-824, Republic of Korea; ^2^Department of Oriental Medicinal Materials & Processing, College of Life Sciences, Kyung Hee University, Global Campus, Gyeonggi 446-701, Republic of Korea; ^3^Graduate School of Biotechnology, Kyung Hee University, Global Campus, Gyeonggi 446-701, Republic of Korea

## Abstract

We devised a study using animal models of hyperthermia and hypothermia and also attempted to accurately assess the effects of *Panax ginseng* (PG) and *Panax quinquefolius* (PQ) on body temperature using these models. In addition, we investigated the effects of PG and PQ in our animal models in high and low temperature environments. The results of our experiments show that mice with normothermia, hyperthermia, and hypothermia maintained their body temperatures after a certain period in accordance with the condition of each animal model. In our experiments of body temperature change in models of normal, low, or high room temperature, the hyperthermic model did not show any body temperature change in either the PG- or PQ-administered group. In the normal and low room temperature models, the group administered PG maintained body temperature, while the body temperature of the PQ-administered group was lower than or similar to that of the control group. In conclusion, the fact that PG increases body temperature could not be verified until now. We also showed that the effect of maintaining body temperature in the PG-administered group was superior in a hypothermia-prone low temperature environment.

## 1. Introduction

Thermoregulation is the ability of an organism to keep its body temperature within certain boundaries, even when the surrounding temperature is very different. The internal thermoregulation process is one aspect of homeostasis: a state of dynamic stability in an organism's internal conditions, maintained far from equilibrium with its environment [1]. Thermoregulation is an important aspect of human homeostasis. High temperatures pose serious stresses for the human body, placing it in great danger of injury or even death [2]. A fall in cellular temperature reduces enzyme efficiency and diffusion capacity, reducing cellular energy availability and membrane ion fluxes. Below-normal brain temperatures are associated with reduced alertness and with behavioural and physiological disorganization, although without physical damage to the CNS or peripheral tissues [3].

*Panax ginseng* Meyer (Korean ginseng) has long been among the most popular botanic products in the world. The major bioactive components of* Panax ginseng* (PG) are saponins, and PG in general is used in tonics and adaptogens to maintain the body's resistance to adverse factors and homeostasis, including enhanced physical and sexual functions, general vitality, and antistress and antiaging functions [4]. In addition, modern pharmacological studies have revealed ginseng's adaptogenic activities against cardiovascular dysfunction and various diseases, including cancer and neurodegenerative disorders [5, 6].

Interestingly, of all the ginseng distributed throughout the world, 90% is in the form of* Panax quinquefolium* Linn., which is also known as American ginseng [7]. In the USA, the effect of ginseng has been advertised as differing from Oriental ginseng (PG) with connotations such as “Unlike Oriental ginseng (PG) known to augment Yang energy,* Panax quinquefolius* (PQ) enhances Yin-energy, and thus its consumption is advocated for people in a hot climate or with hypertension.” Furthermore, PQ has a cooling effect on the body and is preferred for consumption in summer. Further, owing to the mild effect of PQ, it is excellent for women, children, and elderly people [8, 9]. On the other hand, PG raises the body temperature, and thus it is suggested to be inappropriate for people with hypertension or who live in a hot climate. Such claims have been relayed to consumers without any scientific backing. Indeed, recent animal studies have reported results contrary to existing beliefs. In hypothermically stimulated mice, intake of PG leads to a more rapid recovery of body temperature as opposed to control mice, whereas in hyperthermically stimulated mice,* Panax ginseng* fails to increase body temperature compared with that of control mice [10]. Furthermore, in a separate animal model, PG was found to be effective for developing resistance to cooling and helped in stimulating faster recovery from acute hypothermia [11].

Previous studies of animal experiments have assessed body temperature with respect to temperature change of the external environment. This experimental model is limited in that it requires a completely objective assessment of the effect of PG on body temperature. Further, owing to the fact that body temperature measurements in such studies were taken only once after ginseng intake, such measurements fail to show body temperature changes in real time for each environment. Thus, in an effort to more precisely verify the effects of PG and PQ on body temperature, this study of various animal models has assessed body temperature changes in real time for each model. Also, we devised a study using animal models of hyperthermia and hypothermia and also attempted to accurately assess the effects of PG and PQ on body temperature using these models. In addition, we investigated the effects of PG and PQ in our animal models in high and low temperature environments.

## 2. Materials and Methods

### 2.1. Animals

Seven-week-old male ICR mice (Jung-Ang Lab Animal Company) were used in this study. Mice were housed under a 12/12 h light/dark cycle, with food and water provided as needed. All experimental procedures were performed in accordance with the Principles of Laboratory Animal Care (NIH publication, #80-23, revised in 1996) and the Animal Care and Use Guidelines of Nambu University, Korea. During the experiment, mice were fasted, abstaining from both food and water.

### 2.2. PG and PQ Extracts

The extracts of PG and PQ used in this study were obtained from the Department of Oriental Medicinal Materials & Processing and Hanbangbio Laboratory, KyungHee University College of Life Sciences. Dried PG or PQ was purchased from the Kyongdong Oriental Market. PG or PQ was sonicated in 50% ethanol solution for 12 h (three times). Following filtration, the solution was evaporated to dryness in vacuo. Briefly, extracts of PG and PQ were dissolved in distilled water, which was delivered orally to mice at a dose of 300 mg/kg.

### 2.3. Hyperthermic, Normothermic, and Hypothermic Mice

A constant water bath was used to prepare animal models for hyperthermia, normothermia, and hypothermia under anesthesia. Prior to testing, all mice were anesthetized with intramuscular xylazine (0.43 mg/kg) and ketamine (4.57 mg/kg).

**Figure 1:**
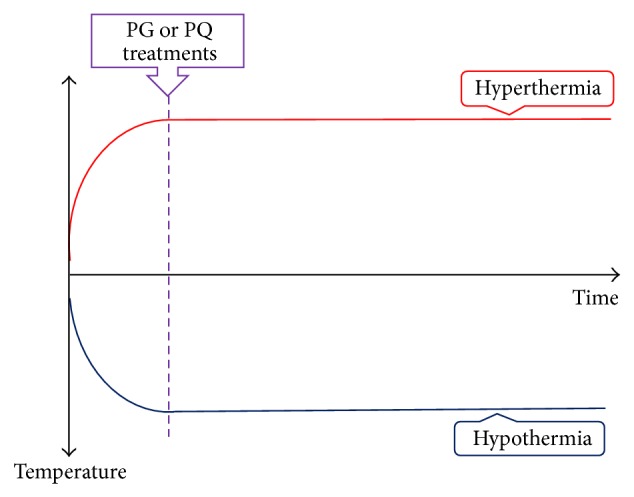
Treatment times for hyperthermia, normal temperature, and hypothermia animal models.

Body temperature was measured rectally using an SDT8A thermometer (Summit Co.) at 10 min intervals. The water bath temperature was set at 38.3°C for establishing hyperthermic animal models. The body temperatures of hyperthermic, normothermic, and hypothermic mice were maintained at 40°C, 37°C, and 34°C, respectively. While establishing these models, body temperature was measured at intervals of 10 min for a total of 130 min. After establishing these models, 8 mice in each of the hyperthermia, normothermia, and hypothermia groups were administered PG and PQ. In the experiment of models of hyperthermia, normothermia, and hypothermia, PG or PQ was administered after 30 min, during which the body temperatures of anesthetized mice were constantly maintained at predetermined conditions (Figure 1).

### 2.4. High and Low Room Temperatures Mice

Temperature-adjustable incubators (HB-103S, HAN BAEK SCIENTIFIC CO., Korea) were used to establish models of high and low room temperatures in unanesthetized mice. An SDT8A thermometer (Summit Co.) was used for the measurement of high, normal, and low room temperature models at 10 min intervals. The high room temperature incubator was kept at 38°C, while the low temperature incubator was maintained at 10°C [10–12]. The normal room temperature was kept at 25°C. After exposing mice to each model of high, normal, or low room temperature for 30 min, PG and PQ were orally administered at one time in eight mice per each group, and body temperature was measured at intervals of ten min for 120 min.

### 2.5. Statistical Analysis

Data were analyzed using Graphpad prism 5 (version 5.01) software (Graphpad Software Inc). All data are expressed as the mean ± S.E.M. Statistical comparisons between groups were performed using two-way repeated measures ANOVAs with Bonferroni post hoc test. *P* values of <0.05, 0.01, and 0.001 were deemed to be statistically significant.

## 3. Results

### 3.1. Establishing Hyperthermic and Hypothermic Animal Models

**Figure 2:**
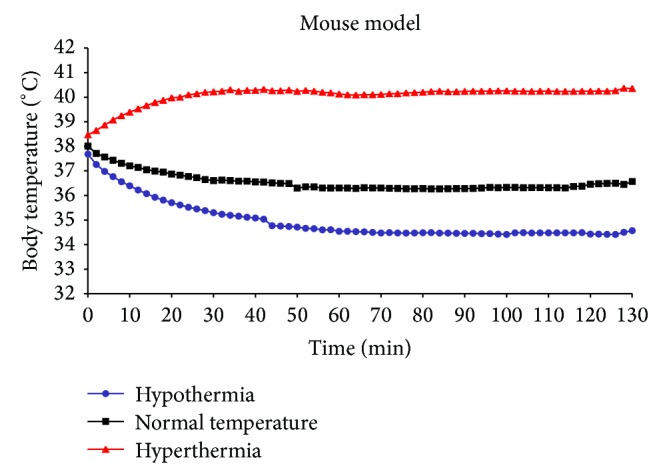
Hyperthermia, normal temperature, and hypothermia mice models. The hyperthermia, hypothermia, and normothermia models were set and maintained at approximately at 40°C, 37°C, and 34.5°C, respectively.

We first established animal models of hyperthermia and hypothermia, which were then used to assess the effect of PG or PQ on body temperature regulation. As shown in Figure 2, the body temperature is dynamic for 60 min. And then, the body temperature is maintained consistently. Hyperthermia, hypothermia, and normothermia models were set and maintained at approximately 40°C, 37°C, and 34.5°C, respectively. PG or PQ was treated at the 30 min after an anesthesia in hyperthermia, hypothermia, and normothermia models because of the reaction time after the treatment and the reduction of an anesthesia stress in mouse.

### 3.2. The Effects of PG and PQ on Body Temperature Regulation of Animal Models of Hyperthermia, Hypothermia, and Normothermia

**Figure 3:**
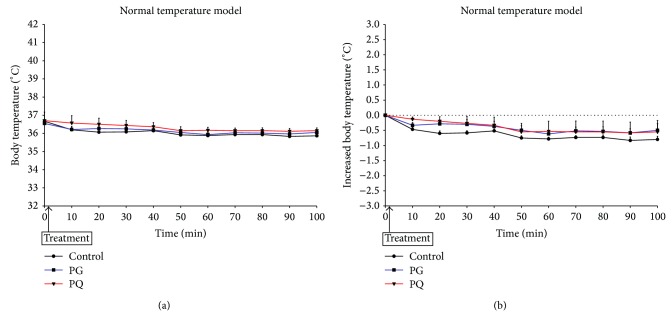
Change in body temperature (a) and increased body temperature (b) in the normal body temperature model. The mice were treated orally at a dose of PG 300 mg/kg (PG), PQ 300 mg/kg (PQ), and distilled water (control).

Figure 3 shows body temperature changes after administration of PG or PQ in a normothermic model. Body temperature was gradually lowered after administration (Figure 3(a)). With respect to the extent of body temperature change, the body temperature of PG- or PQ-administered animals exhibited a somewhat small drop in temperature compared with the control group, although the difference was not significant. Likewise, there were no significant differences in the degrees of body temperature change between PG and PQ groups (Figure 3(b)).

**Figure 4:**
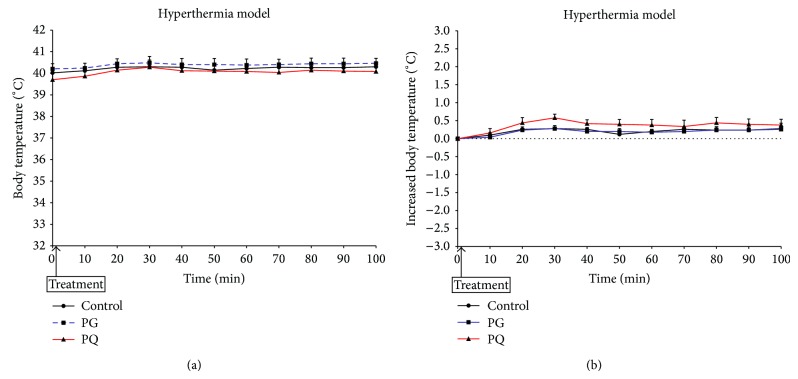
Change in body temperature (a) and increased body temperature (b) of hyperthermia model. The mice were treated orally at a dose of PG 300 mg/kg (PG), PQ 300 mg/kg (PQ), and distilled water (control).

Figure 4 shows the body temperature change after administration of PG or PQ in the hyperthermic model. The body temperatures of all groups were constantly maintained after administration of samples (Figure 4(a)). The body temperature change of the PG-administered group was similar to that of the control group. However, the body temperature change of the PQ-administered group was somewhat increased as opposed to that of the control group, although the difference was not significant (Figure 4(b)).

**Figure 5:**
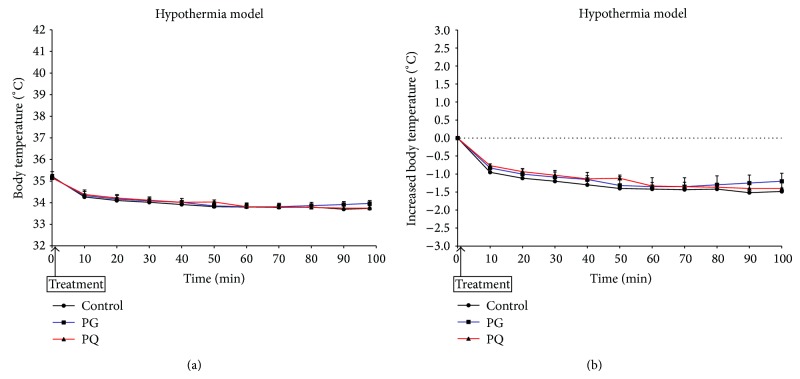
Change in body temperature (a) and increased body temperature (b) in the animal model of hypothermia. The mice were treated orally at a dose of PG 300 mg/kg (PG), PQ 300 mg/kg (PQ), and distilled water (control).

Figure 5 shows body temperature change after PG or PQ administration in the hypothermic model. The body temperatures in all groups were gradually decreased for 60 min after sample administration and then maintained (Figure 5(a)). The body temperature changes of PG and PQ administered groups, as well as that of the control group, were similarly maintained (Figure 5(b)).

### 3.3. The Effects of PG and PQ on Body Temperature Regulation in Animal Models at Normal, High, and Low Room Temperature

**Figure 6:**
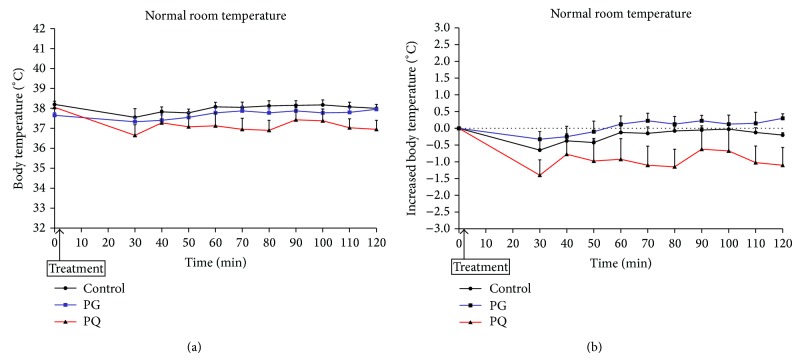
Change in body temperature (a) and increased body temperature (b) of normal room temperature model. The mice were treated orally at a dose of PG 300 mg/kg (PG), PQ 300 mg/kg (PQ), and distilled water (control).

Figure 6 shows the changes in body temperature after administration of PG and PQ in the normal room temperature model. The body temperatures in control and PG treatment groups were constantly maintained. The body temperatures in PQ treatment group were lowered for 30 min after administration and then maintained (Figure 6(a)). The body temperature of the PG-administered group and that of the control group were maintained at a level similar to that of the early phase. However, the body temperature of the PQ administered group was maintained at a level lower than that of the early phase. However, the difference of the PQ administered group was not significantly different from that of the PG administered group or the control group (Figure 6(b)).

**Figure 7:**
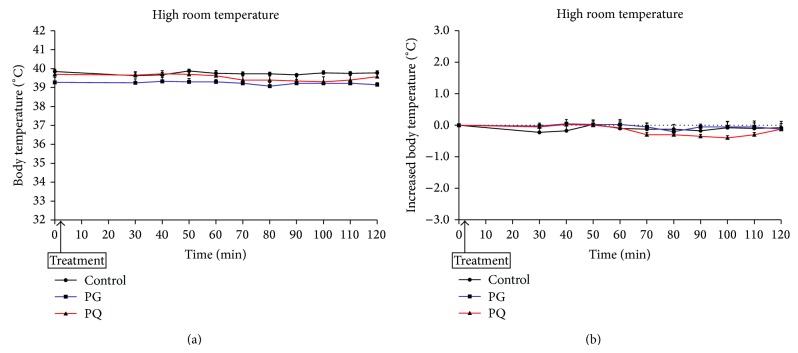
Change in body temperature (a) and increased body temperature (b) of high room temperature model. The mice were treated orally at a dose of PG 300 mg/kg (PG), PQ 300 mg/kg (PQ), and distilled water (control).

Figure 7 shows the changes in body temperature in the high room temperature model after administration of PG or PQ. Body temperatures of all groups were steadily maintained (Figure 7(a)). The body temperatures of the sample-administered groups and control group were maintained at a level similar to that of the early phase (Figure 7(b)).

**Figure 8:**
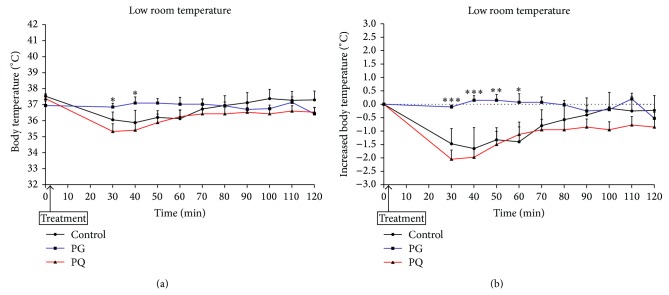
Change in body temperature (a) and increased body temperature (b) of the low room temperature model (^*^ *P* < 0.05, ^**^ *P* < 0.01, and ^***^ *P* < 0.001 versus PQ). The mice were treated orally at a dose of PG 300 mg/kg (PG), PQ 300 mg/kg (PQ), and distilled water (control).

Figure 8 shows the changes in body temperature of the low room temperature model after PG or PQ administration. The body temperature of the PQ administered group and control group decreased up to 30 min after sample administration and increased thereafter. The body temperature of the PG-administered group was maintained throughout the test period. There was a significant difference in the body temperatures of the PG administered group at 30 and 40 min after sample administration compared with the control group (Figure 8(a)). With respect to changes in body temperature, the control group gradually decreased for 30 min after sample administration, after which it gradually increased until 100 min after administration, making a full recovery to the initial body temperature.

The body temperature of the PQ-administered group also decreased gradually for 30 min after sample administration, after which it slowly increased until 70 min after administration. However, after 70 min, the body temperature decreased and was maintained at a level lower than that of the early phase. The body temperatures of the PG-administered group were maintained at a level similar to that of the early phase. There were significant differences in the body temperature changes between the PG-administered group and PQ-administered group at 30, 40, 50, and 60 min after administration (^*^ *P* < 0.05, ^**^ *P* < 0.01, and ^***^ *P* < 0.001 versus PQ) (Figure 8(b)).

## 4. Discussion

This study assessed the effects of PG and PQ on body temperature regulation in hyperthermic and hypothermic animal models. We also examined the effects of PG and PQ on body temperature changes upon exposure of animals to high and low ambient temperatures. Hyperthermic, hypothermic, and normothermic animal models were subjected to increased or decreased body temperature after induction of anesthesia. In addition, a steady body temperature was maintained before PG and PQ were administered in order to assess the effect of PG or PQ administration on body temperature regulation under controlled conditions. In addition, the high, low, and normal room temperature models allowed us to prepare an environment in which the body temperature could change, as well as to evaluate the time-dependent effect of PG and PQ administration on body temperature regulation while controlling for external factors, such as environment.

Body temperature is controlled by the thermoregulatory center in the hypothalamus. It receives input from two sets of thermoreceptors: receptors in the hypothalamus itself monitor the temperature of the blood as it passes through the brain, and receptors in the skin monitor the external temperature. Both sets of information are needed so that the body can make appropriate adjustments. The thermoregulatory centre sends impulses to several different effectors to adjust body temperature. This is part of the autonomic nervous system, so the various responses are all involuntary [13]. Also, factors to regulate body temperature may affect body temperature, secondary to the environment. For instance, hypothermia induces dynamic muscular activities to increase body temperature. Hyperthermic, hypothermic, and normothermic animal models were developed to be excluded from secondary activity for body temperature regulating.

High and low ambient temperatures models have been utilized in a number of studies that have evaluated the effect of ginseng on body temperature [11, 14]. The animal models in previous studies have been subjected to administration of ginseng over a five-day period, during which mice were exposed to different surrounding temperatures, with the body temperature being measured only at the end of the study. In the present study, we evaluated patterns of body temperature by continuously measuring temperature at 10 min intervals at varying ambient temperatures. In a study of body temperature using different environmental temperatures, factors other than medicinal substances administered to regulate body temperature may affect body temperature, secondary to the environment. For instance, hypothermia induces dynamic muscular activities to increase body temperature. Likewise, hyperthermia may cause the body to react in such a way as to decrease body temperature by excretion or exhalation. Thus, our latter models were limited in their ability to assess the efficacy of body temperature regulation by PG or PQ.

The results of our experiments show that mice with normothermia, hyperthermia, and hypothermia maintained their body temperatures after a certain period in accordance with the condition of each animal model. There were no differences in body temperature between sample groups and the control group, even in cases where body temperatures increased and decreased. Furthermore, there were no differences in changes to body temperature between PG- and PQ-administered groups. Thus, in our animal models utilizing different body temperatures, administration of PG or PQ did not affect the induced changes in body temperature.

In our experiments of body temperature change in models of normal, low or high room temperature model did not show any body temperature change in either the PG-administered group, while body temperatures of PQ-administered groups were lower than the control group or similar to that of the control group. And recovery of the initial body temperature was not attained within the 120 min measurement period. Thus, our results showed that PQ was not helpful in maintaining the body temperature in each environment, while PG-administration maintained body temperature in ambient and hypothermic conditions in a helpful manner. These results contrast with the common misunderstanding that PG often increases body temperature, while PQ decreases body temperature, thus indicating that PG is inappropriate for individuals with a tendency for hyperthermia. Indeed, our data suggest that PG may be indicated for both maintaining body temperature and inhibiting a decline in body temperature.

The effect of maintaining the body temperature in PG-administration mice might be related to the differences between ginsenosides of PQ and PG. Major ginsenosides of PG are Rb1, Rg1, and Rb2; otherwise, PQ have Rb1, Re, and Rd [15]. In addition, the ratio of Rg1/Rb1 has been widely used to differentiate between these ginsengs. Ratios of less than 0.4 are indicative of PQ, whereas a high value ratio is characteristic of PG [16]. In the previous report, the infusion of ginsenoside Rg1 into the rat maintained the rectal temperature in ambient temperature changes [17]. We should suggest that a high value ratio of PG is the important clue of the effect of the maintained body temperature.

The thermoregulatory effect of PG is confirmed only in environmental condition, normal and low room temperature models. Environmental condition models included factors other than independently PG or PQ efficacy to regulate body temperature. We might be suggesting that the thermoregulatory effect of PG in those models included the interaction effect between PG treatment and secondary activity.

A previous study which evaluated the recovery process of body temperature after thermovariations in animal models reported that both PG and PQ were helpful in maintaining body temperature in high and low temperature environments [11]. The present study assessed the pattern by which body temperatures changed in real time. It was ascertained that body temperature would be maintained by the prevention of the early phase decline of body temperature.

A previous study evaluated the effect of PG-administration on animals exposed to high ambient temperatures by measuring its effect on body temperature. In that study, exposure of mice to a high temperature environment of 38°C for five hours a day for seven days led to decreased weight gain, increased white blood cell count, and increased levels of hepatic malonic dialdehyde (MDA). On the other hand, the rate of weight gain in mice exposed to high ambient temperatures and administered PG was similar to that of the mice that were not exposed to a high temperature environment. Further, these mice had white blood cell counts within normal levels and also a suppression of the increase in hepatic MDA content [12]. Conversely, a clinical study conducted in Korea reported that PG administration had no effect on body temperature as determined by infrared measurements [18].

In general, PG and PQ have a good safety record. The root of PG and PQ appeared nontoxic to rats, dogs, and humans [19]. In mice, a lethal oral dose of purified ginseng was determined to be higher than 5 g/kg, the highest dose that can be orally given to a mouse and considered good practice at the maximal dose volumes without violating animal welfare standards [20]. In human study, PG administration for 4 weeks was shown to be safe, tolerable, and free of any untoward toxic effect in healthy male and female volunteers [21]. It is well known that the most common side effects of PG or PQ resulting from its overdose are nervousness and excitability. These side effects usually decrease after the first few days.

In this study, we used the only one dose of PG and PQ. We selected the one dose of 300 mg/kg in PG or PQ treatments because previous reports in metabolic studies are used to this dose, a dose which showed the significant difference in metabolic disease animal model [22, 23]. Also, when we performed the preliminary tests using the 300 mg/kg in PG or PQ treatments in mouse, finally we selected 300 mg/kg dose. We think that it is necessary to investigate different doses in further study.

In the present study, the common understanding that PG increases body temperature could not be verified using two separate animal models. We also showed that the effect of maintaining body temperature in the PG-administered group was superior in a hypothermia-prone low temperature environment, suggesting that PG intake may be helpful in maintaining a consistent body temperature in animals. Studies on the mechanism by which PG maintains body temperature have yet to be reported. Therefore, evaluation of this mechanism should be attempted through future studies relating to maintenance of body temperature.
